# Reading and Oral Language Skills in Children With Developmental Language Disorder: Influence of Socioeconomic, Educational, and Family Variables

**DOI:** 10.3389/fpsyg.2021.718988

**Published:** 2021-10-08

**Authors:** María Fernanda Lara-Díaz, Angélica Mateus-Moreno, Judy Costanza Beltrán-Rojas

**Affiliations:** Department of Human Communication, Faculty of Medicine, Universidad Nacional de Colombia, Bogotá, Colombia

**Keywords:** developmental language disorder, reading, socioeconomic status, vocabulary, reading comprehension

## Abstract

The Developmental Language Disorder (DLD) is a delay in language skills that cannot be explained by sensory or cognitive difficulties. Currently, there are limited studies that analyze how socioeconomic, educational, and family variables influence reading skills of Spanish-speaking children with DLD at school. This study identifies how oral language performance and reading skills of children with DLD are linked to socioeconomic, educational, and family factors. Oral language, phonological awareness and reading abilities were assessed in a sample of 15 children diagnosed with DLD and their controls by age and gender. Children's parents answered a Likert scale questionnaire inquiring about some aspects related to the family's socioeconomic status, mothers' educational level, family support, academic average, and repetition of school years of the participants. The results indicate that children with DLD have a lower performance in phonological awareness tasks as well as in reading abilities. There is also a direct relationship between their performance in language and reading skills and variables as mother's educational level and family support. Likewise, children in the sample have a lower academic average as well as a higher school year repetition rate interfering in their academic life. Educational implications of these findings and a discussion on possible causality axes and protective factors that contributes to support this population are presented.

## Introduction

The Developmental Language Disorder (DLD) is a neurodevelopmental condition affecting the development of communicative skills in children who demonstrate normal non-verbal intelligence and do not present hearing, visual or environmental impairments which explains the disorder (Bishop and Hayiou-Thomas, 2008). The definition, scope, and characteristics of DLD have been defined in consensus by experts on the subject since 2016 (Bishop et al., 2017). Consequently, public awareness, identification of cases, and access to therapeutic services and educational support have improved (McGregor et al., 2020). However, in Colombia there are neither epidemiological data of the incidence of DLD in children nor on the access of these children to therapeutic or educational services.

DLD affects several linguistic levels and processes, such as morphosyntax (Conti-Ramsden et al., 2001), phonological processing (Montgomery and Windsor, 2007; Claessen et al., 2013; Aguilar-Mediavilla et al., 2014), reading (Bishop and Snowling, 2004; Pennington and Bishop, 2009) and writing (Graham et al., 2020), as well as cognitive skills as processing speed (Miller et al., 2001), auditory attention (Ebert et al., 2019), executive functioning (Pauls and Archibald, 2016), and working memory (Ghandour et al., 2018). Moreover, there are alterations related to behavior and emotional regulation transcending past childhood (Conti-Ramsden et al., 2019).

It has been determined that this disorder prevails in school-age children, as approximately between 7 and 8% of this population suffer from DLD (Tomblin et al., 1997). There is no precise data on its incidence and prevalence in Colombia; thus, it is necessary to give this condition a more sample scope in as much as it has a significant impact on social interactions and academic performance of children with DLD (Bishop et al., 2017).

Socioeconomic level has been documented as a factor of great influence in language disorders (Auza-Benavides et al., 2019). Parents level of education and occupation are related to children's linguistic performance in vocabulary and grammar as well as in their cognitive measurements. Thus, socioeconomic status should be considered as a longitudinal intervening factor (Gathercole et al., 2016). In the specific case of Colombia, due to the adaptation of the MacArthur Bates Communicative Development Inventories (CDI), an instrument for evaluating linguistic development, the educational level of the mother is associated with vocabulary repertoire of children, whereas economic income has no significance; this is explained by the regional disparity in this relationship (Lara Díaz et al., 2011).

It has been broadly described how children with speech and language difficulties have a greater risk of having difficulties in their literacy process (Oliveira et al., 2021), making errors during the learning process to read and to write (Hulme and Snowling, 2016), and showing deterioration in phonological processing tasks (Catts et al., 2005). The last aspect is, in fact, a predictor for academic skills. Bishop and Snowling (2004) suggest that dyslexia and DLD are characterized by a deficient phonology, that becomes a risk factor in reading decoding processes; however, they differ in language measurements such as vocabulary and comprehension.

With respect to reading acquisition, Tomblin et al. (2000) found that most of the children that had language impairments also demonstrated difficulties in reading accuracy of words and pseudo-words as well as a low performance in reading comprehension. Reading comprehension shares the same processes involved in oral comprehension (Hjetland et al., 2019; Nation, 2019). Consequently, if the processes needed for oral language are not working correctly, reading comprehension will be also affected. Different studies have found the same correlation (Lervåg et al., 2018).

Nonetheless, profiles in language and reading difficulties differ within different languages (Bialystok et al., 2005). As a result of the differences between opaque and transparent spellings, it is necessary to carry out separate studies for different languages. Spanish language has a transparent orthography, as there is a closer relationship between graphemes and phonemes. Therefore, evidence obtained from other languages, such as English, is not totally transferable to Spanish speakers' contexts. Determining the role and weight of the phonological components in reading is a necessary task. Research about English language has found that children with DLD show comprehension difficulties and lower reading accuracy (Snowling et al., 2019). In Spanish-speaking contexts, children with DLD require less phonological knowledge due to the language's spelling transparency. This should, theoretically, contribute to the learning process of reading and writing; nonetheless, these children tend to present a high percentage of reading accuracy impairments and comprehension difficulties (Pratt et al., 2020).

In the reading process, the relationship can be linked to the “Matthew Effect,” a model that describes the cumulative advantages of educational results. Thus, an initial advantage leads to good results tending to produce new advantages. In contrast, an initial disadvantage generates a more disadvantageous situation (Stanovich, 2009; Rigney, 2010) which creates a growing gap. This cumulative effect is evidenced in the correlation between language impairments and the development of successive reading skills. Diuk and Ferroni (2012) have approached this phenomenon in Spanish language and linked poor reading skills to unfavorable socioeconomic conditions.

These disadvantages are frequently reflected in their academic performance. Studies in Spanish and Catalan speaking children have found that children with DLD have a higher tendency to repeat a school year and obtain significantly lower grades in all cycles and all subjects in comparison with their classmates (Aguilar-Mediavilla et al., 2019). Furthermore, language impairments have been identified as risk factors that change the course of the expected development and have consequences transcending childhood such as higher probability of aggressiveness and delinquency (Brownlie et al., 2004), lower income, and less work opportunities. Those factors have a significant impact not only on academic performance but also on general well-being (Records et al., 1992).

Despite the serious consequences of DLD in children's development there are not enough studies that analyze the correlation between socioeconomic, educational, and family variables in Spanish speaking children with DLD and with the development of reading skills at school. The aim of this study is to identify how oral language difficulties and reading skills in children with DLD are linked to socioeconomic, educational, and family factors; thereby, this research contributes to the understanding of DLD in Spanish, considering that Spanish differs from other languages such as English in terms of the transparency on its spelling which has repercussions on the difficulties that children may face during their learning processes.

## Methodology

### Participants

This study involved 30 Spanish speaking Colombian children aged between 9 and 12 years old. There were 15 children with DLD in the study group and 15 children without language difficulties in the control group. All participants were enrolled in school and spoke only Spanish. The control group was selected having the same age and gender characteristics of the study group (both groups had the same male:female ratio: 11 men:4 women). In Colombia, the educational system is organized in four levels: initial education, preschool education, basic education (elementary school from grades 1° to 5° and secondary from grades 6° to 9°), middle education (grades 9° to 11°), and higher education (Ministerio de Educación Nacional, 2019). All participants were in elementary school (3°-5° grade) and were matched by age and gender, but not by grade in all cases, since there were children with DLD who had repeated courses, so they were one or two courses behind their age peers.

Participants were selected from public and private schools. In Colombia, public schools have a greater number of students than those ones who do not have the economic resources to afford a private school. On the other hand, private schools in Colombia have a lower number of students per classroom, as well as greater monitoring of their educational process since they have different support professionals such as speech therapists, occupational therapists, and psychologists.

Table 1 shows the sociodemographic data of the participants. Speech therapists diagnosed study group participants with a language delay and their contacts were provided by schools and therapists. From this first referral, the participants were evaluated to verify compliance with the inclusion criteria based on the language evaluation battery and the non-verbal intelligence test (see Procedure section). The inclusion criteria required that none of the children had other conditions explaining the language deficits, such as autism, hearing loss, or intellectual disability. Each participant was assessed with a language assessment battery to confirm difficulties in this area.

**Table 1:** Sociodemographic data of participants.

	**DLD group**	**Control group**	**Mann Whitney U**	***p***	***Z***
Age	10.4 (1.1)	10.4 (1.1)	112.5	1.0	0.00
**Gender**				
Female	4	4	112.5	1.0	0.00
Male	11	11	112.5	1.0	0.00
School grade	3.67 (0.9)	4.53 (1.3)	67.99	0.61	−1.95
K-BIT	92.4 (2.9)	94.3 (9.6)	85.0	0.267	−1.14

### Instruments and Materials

Each participant answered the non-verbal intelligence test from the Kaufman Brief Intelligence Test (K-BIT) (Kaufman, 1997), which evaluates the non-verbal intelligence coefficient from the matrix test. Receptive and expressive language levels were assessed with the Spanish Clinical Evaluation of Language Fundamentals 4 (CELF-4) battery (Semel et al., 2004). Particularly, there are four subtests of the first level of the CELF-4 that make up the score for core language. This score is used as a screening for clinical decision making in this age range (Andersson et al., 2019). The four tests applied in this stage are concepts and following directions, recalling sentences, word classes, and formulated sentences. From these subtests, the receptive, expressive and core language scores were measured.

The vocabulary variable was assessed through the Peabody Vocabulary Test (PVT), which was adapted for Latin American population in 1986 (Dunn et al., 1986). Various studies have suggested that children with DLD are at risk of presenting difficulties in their literacy process due to impairments in phonological awareness (Zourou et al., 2010); for this reason, this skill was assessed by the Evaluation of Phonological Processing (PROFON) (Lara et al., 2007). Three levels of phonological awareness were evaluated with this battery: syllabic, intrasyllabic, and phonemic. Finally, the reading level of the participants was assessed with the Revised Evaluation Battery of Reading Processes (PROLEC-R) (Cuetos et al., 2007).

An *ad-hoc* questionnaire, developed for this study, was applied to children's parents. The answers provided data regarding socioeconomic aspects and status, educational levels, availability of family support. In Colombia, socioeconomic status is determined by the area where families live and their monthly income (from 1 to 6, with 1 being the lowest category). For the analysis of the study, this variable was categorized according to the following scale: low: 1 y 2 stratum, medium:3 y 4 stratum, high: 5 y 6 stratum.

The educational level of the mother was considered using the following scale: elementary: 1, high school: 2 technical: 3, professional: 4, postgraduate: 5. The level of support that each participant received from his/her parents regarding academic activities was indicated in the survey through examples to clarify the question. Family support was understood as the support they gave their children in their homework time, the regularity of parents' attendance to school meetings, amount of time they spent with their children in activities non related with schoolwork, among other aspects related to the role of primary caregivers. This parameter was also analyzed through a Likert scale.

Within educational variables, questions included grade average rated with a Likert scale from 1 to 5, the type of school he/she attended to (public:1, private:2), and the number of grades he/she had repeated, as well as sociodemographic data such as age and current school grade.

### Procedures

This study was developed in three phases. In the first phase, participants with DLD were recruited with a direct invitation sent to institutions and speech language therapists. During the second one, language and non-verbal intelligence tasks were applied to verify that the participants fulfilled the inclusion criteria for the study. In the third stage, the participating children completed reading and vocabulary tests, and their parents answered the questionnaire.

The study was carried out in the cognitive neuroscience and communication laboratory of National University of Colombia and the tests were done by trained speech therapists and psychologists. No financial compensation was made to the participants, but the results report was given to the participants and their families, and a referral was made to a speech therapy or psychology service if the participant required it. This study was approved by the Research Ethics Committee of the National University's Faculty of Medicine. Additionally, parents signed an informed consent and children agreed to participate as well.

### Data Analysis

Data analysis and treatment were performed using the version 22.0 of the statistical software SPSS. Non-parametric statistics were applied. The Mann-Whitney (*U*) test was used after the evaluation of assumptions through the Shapiro-Wills and Kolmogorov- Smirnov tests. Comparisons of means, differences of means, and analysis of variance were performed to compare the control and study groups. A significance level of *p* < 0.05 was applied for all comparisons. Non-parametric correlations were calculated using the Spearman correlation coefficient between the main variables of the study. Multiple linear regression models were generated to explain the dependent variables: reading accuracy and reading comprehension from independent variables, those were related to language skills and socio-economic and educational skills from the correlations made in the previous step. A successive step linear regression model was used to explore the interaction between different independent variables in relation to the reading level of the participants.

## Results

### Headings Language Level: Receptive and Expressive

The analysis of results of language tasks was performed identifying statistically significant differences between the study and the control groups. In general, the DLD group had a lower performance in all the tasks. The higher impairment was observed on the word class task which has a receptive and an expressive component, and on the formulated sentences task, in which children must build a sentence from a given image. These flaws are related to the expected profile of children with DLD, who are characterized by impairments in the morphosyntactic component (Leonard et al., 1992). Moreover, comprehension and expressive levels were below the expected average for the participants' age. Table 2 details the results of the assessed tasks. In line with these findings, the PVT vocabulary tasks also found a significant difference between the DLD group and the control group.

**Table 2:** Language tests results.

**Language tasks**		**DLD group**	**Control group**	**Mann Whitney U**	***p***	***Z***
CELF-4	C and FD	30.3 (3.5)	43.9 (7.9)	0.00	0.00 *	−4.683
	RS	49.6 (7.9)	78.1 (6.4)	1.00	0.00*	−4.629
	WC-total	22.1 (4.3)	36.9 (8.5)	15.00	0.00*	−4.052
	FS	23.6 (7.2)	44.8 (4)	0.50	0.00*	−4.655
	Core language	25.4 (2.9)	46.1 (4.9)	0.00	0.00*	−4.685
	Receptive language	11.2 (1.4)	21.2 (2.6)	0.00	0.00*	−4.686
	Expressive language	21.2 (2.6)	35.8 (3.7)	0.00	0.00*	−4.677
PVT		74.2 (12.3)	100 (8.3)	10.00	0.00*	−4.258

*C and FD, concepts and following directions; RS, recalling sentences; WC-Total, Word Class-total; FS, formulated sentences; PVT, peabody vocabulary test*.

* *The correlation is significant at the 0.05 level (bilaterally)*.

**The correlation is significant at the 0.01 level (bilaterally).

### Performance in Phonological Awareness Tasks

Phonological awareness performance was assessed with PROFON test (Lara et al., 2007). The DLD group had a lower performance in all phonological awareness levels (syllabic, intrasyllabic, and phonemic). At the phonemic level the ability to segment and synthesize phonemes is evaluated and related to the degree of sensitivity to the language's sound structure. This ability has a strong connection to reading acquisition (Anthony and Francis, 2005), especially in Spanish (Goldenberg et al., 2014). The results of the study group in this level shows a significantly lower performance in comparison with the control group, as described in Table 3. These results are related to the findings suggesting that children with DLD may present phonological awareness deficits, which impact their subsequent literacy processes (Zourou et al., 2010).

**Table 3:** Phonological awareness results.

**Phonological awareness levels**	**DLD group**	**Control group**	**Mann Whitney U**	***p***	***Z***
Syllabic level	10.4 (2.3)	14.4 (0.8)	14.50	0.00*	−4.146
Intrasyllabic level	8.27 (3.7)	17.6 (1.9)	5.00	0.00*	−4.475
Phonemic level	5.27 (1.6)	9.07 (0.79)	2.00	0.00*	−4.636
Total	23.9 (6.7)	41.1 (2.9)	4.50	000*	−4.490

* *The correlation is significant at the 0.05 level (bilaterally)*.

**The correlation is significant at the 0.01 level (bilaterally).

### Reading Tasks Performance: Accuracy, Comprehension, and Speed

Reading is an essential skill for schooling. It can determine the educational success or failure of a person since most of the knowledge is transmitted through written material (Küçükoglu, 2013). In that way, in this study reading is a crucial factor to determine DLD's impact on participants' school life. Table 4 describes how the study group had a significantly lower performance for the reading skills assessed in letter, word, pseudo-word, and text levels. These difficulties were evidenced in the areas of comprehension, accuracy, and speed.

**Table 4:** PROLEC results.

**Reading tasks-PROLEC-R**	**DLD group**	**Control group**	**Mann Whitney U**	***p***	***Z***
Letter identification (a)	16.5 (3.0)	19.7 (0.5)	20.00	0.00*	−4.034
Letter identification (t)	28.7 (14)	16 (3.7)	38.50	0.002*	−3.077
Words reading (a)	23.9 (11.4)	39 (1.1)	7.50	0.00*	−4.396
Words reading (t)	162.4 (102)	41.2 (14.2)	6.00	0.00*	−4.420
Pseudowords reading (a)	19.5 (10.3)	37.5 (2.2)	1.50	0.00*	−4.614
Pseudowords reading (t)	183.1 (116)	64.6 (17)	1.50	0.00*	−4.111
Text reading (c)	1.73 (1.3)	3.53 (0.64)	29.00	0.00*	−3.606
Text reading (t)	215.2 (127)	53.6 (20.3)	4.00	0.00*	−4.501

*a, accuracy; t, time; c, comprehension*.

* *The correlation is significant at the 0.05 level (bilaterally)*.

**The correlation is significant at the 0.01 level (bilaterally).

### Characterization of Socioeconomic, Family and Educational Variables

The differences between both groups were contrasted with socioeconomic, family, and educational variables found in the parents' questionnaire. High effect were found for variables such as the mother's educational level (*U* = 12, *p* = 0.0; *Z* = −4.33), family support (*U* = 59.5, *p* = 0.01; *Z* = −2.635), academic grade point average (*U* = 20.0, *p* = 0.0; *Z* = −4.060), and the repetition factor (*U* = 37.5, *p* = 0. 0; *Z* = −3.78). These data suggest that children with DLD tend to present a lower academic average at school along with a higher rate of grade repetition. This trend can be directly linked to the flaws found in reading performance and oral language level, which interfere with effective literacy processes. Socioeconomic status, the type of school (private:2 or public:1), and school grade showed no significant differences between groups (Table 5).

**Table 5:** Socioeconomic, family, and educational variables.

	**DLD group**	**Control group**	**Mann Whitney** ***U***	***p***	***Z***
Socioeconomic status	2,4 (0.7)	2.93 (0.5)	71.5	0.08	−1.944
Mother's educational level	1.93 (0.79)	3.60 (0.5)	12.0	0.00*	−4.33
Family support	2.53 (1,45)	3.87 (0.35)	59.5	0.01*	−2.635
School type	1.60 (0.50)	1.53 (0.51)	105.0	0.71	−0.362
School grade	3.67 (0.9)	4.53 (1.3)	67.99	0.61	−1.95
Academic grade point average	1.53 (0.51)	2.87 (0.74)	20.0	0.00*	−4.060
Repetition factor	0.73 (0.59)	0.0 (0.0)	37.5	0.00*	−3.780

* *The correlation is significant at the 0.05 level (bilaterally)*.

**The correlation is significant at the 0.01 level (bilaterally).

### Correlation Between Socioeconomic, Family, and Educational Variables and Language, Phonological Awareness and Reading Skills

Non-parametric correlations were made, and they indicate that variables such as socioeconomic status, educational level of the mother, family support, and academic average have significant correlations with most of the language, reading and phonological awareness skills. There is a greater effect size between the mother's educational level and the language and reading skills. Socioeconomic status has a smaller effect in all the tasks. Academic grade point average has significant correlations with literacy skills (oral language and reading) at school as shown in Table 6.

**Table 6:** Correlation matrix: socioeconomic, family and linguistic variables.

	**Language level**				**Phonological skills**	**Reading**		
	**Core**	**Receptive**	**Expressive**	**Vocabulary**	**Phonological awareness**	**Words**	**Pseudo-words**	**Text**
SES	0.480**	0.482**	0.480**	0.578**	0.522**	0.316	0.379*	0.154
MEL	0.828**	0.770*	0.845**	0.700**	0.809**	0.739**	0.739**	0.445*
FS	0.673**	0.260	0.674**	0.658**	0.,598**	0.559**	0.621**	−0.241
Academic average	0.753**	0.759**	0.754**	0.,697**	0.616**	0.586**	0.650**	0.468**

*SES, socioeconomic status; MEL, mother's educational level; FS, family support*.

* *The correlation is significant at the 0.05 level (bilaterally)*.

** *The correlation is significant at the 0.01 level (bilaterally)*.

### Relationship With Subsequent Literacy Skills: Reading Accuracy and Comprehension

After determining the strength of the association among the variables of the study, a description of their relationship was developed through successive step lineal regression models. These models allowed to demonstrate how oral language; children's performance in literacy processes such as accuracy and reading comprehension, socioeconomic, family, and educational variables are linked. Additionally, possible protective or risk factors immersed in these relationships were determined.

With respect to reading accuracy and phonological awareness skills, the phonemic level is specifically related to the pseudo-word reading task, predicting 60% of variance (*F* = 44). Analyzing the impact of socioeconomic, family, and educational variables on reading accuracy, it was found that the mother's educational level predicts about 50% of variance (*F* = 27). However, when the variable of family support received by the participant is considered with to the mother's educational level, it can be predicted up to 57% of results in reading accuracy (*F* = 17). The socioeconomic status and the repetition level did not show a significant association for the models presented (Table 7).

**Table 7:** Linear regressions: reading accuracy.

**Variable**	***F***	***p***	***R*** **^2^**	**Δ***R***^2^**
**Language skills**				
Formulating sentences	*F*_(1.28)_ = 38.83	0.00	0.58	0.56
Phonological awareness skills	*F*_(1.28)_ = 44.94	0.00	0.61	0.60
Vocabulary level	*F*_(1.28)_ = 16.01	0.00	0.36	0.34
**Socioeconomic, family, and educational variable**				
Mother's educational level	*F*_(1.28)_ = 27.01	0.00	0.49	0.47
Family support	*F*_(1.28)_ = 23.24	0.00	0.45	0.43
Socioeconomic status	*F*_(1.28)_ = 5.95	0.21	0.17	0.14
Repetition level	*F*_(1.28)_ = 5.53	0.26	0.16	0.13

In terms of reading comprehension, as a dependent variable, and the formulating sentences subtest as a predictor variable, it was found that this task predicts 50% of the variance in reading comprehension (*F* = 30) (Table 8). This is an oral language task where participants must structure a sentence according to visual input. This relationship is interesting since the persistent flaws at the morphosyntactic level presented in children with DLD affect not only accuracy, but also reading comprehension processes. After analyzing whether there are other factors that predict the behavior of the data in relation to reading comprehension, it was found that only the educational level of the mother can predict 21% of the variance (*F* = 8.9), showing a low effect in the linear relationship of the variables analyzed, which emphasizes how the linguistic level has a greater relationship with the later literacy results.

**Table 8:** Linear regressions: reading comprehension.

**Variable**	***F***	***p***	***R*** **^2^**	**Δ***R***^2^**
**Language skills**				
Formulating sentences	*F*_(1.28)_ = 30.11	0.00	0.51	0.50
Phonological awareness skills	*F*_(1.28)_ = 19.86	0.00	0.41	0.39
Vocabulary level	*F*_(1.28)_ = 7.64	0.10	0.21	0.18
**Socioeconomic, family, and educational variable**				
Mother's educational level	*F*_(1.28)_ = 8.92	0.06	0.24	0.21
Repetition level	*F*_(1.28)_ = 8.62	0.07	0.23	0.20

From the variables analyzed, it is found that the level of language (core language) explains around 63% of the variance in relation to the academic average of the participants (*F* = 51). This fact explains why language is a fundamental axis during the schooling and why those children with DLD will present greater difficulties in their subsequent literacy processes.

## Discussion

This study intends to identify how oral language performance and reading skills of Spanish speaking children with DLD are related to socioeconomic, family, and educational factors. Findings demonstrate that children with DLD have a lower performance in all reading tasks, especially in accuracy, comprehension, and phonological awareness, compared to the control group. Moreover, results suggest that the mother's academic level and family support are strong factors related to children's linguistic performance.

Students with DLD and reading difficulties have problems with phonological processing. This difficulty puts them at high risk of having poor reading skills (Snowling et al., 2019). Some researchers propose that the common base is deficiencies in phonological processing, affecting the development of language and the acquisition of reading skills such as the acquisition of the alphabetic principle, reading of new words, comprehension of texts, reading fluency and phonological memory (Catts and Kamhi, 2005; Scarborough, 2005).

In this study, phonological awareness skills in the phonemic level predicted more than 50% of results in word reading. This income matches with the theory that states that phonological awareness skills at the intrasyllabic level are better predictors for reading in opaque spellings such as English; however, phonemic skills, as well as phoneme-grapheme correspondence have more influence on reading accuracy tasks, such as pseudo-word reading, in more transparent spellings as Spanish (Kim and Pallante, 2012). In that way, this study has found a stronger relationship, between phonological skills and reading of new words and between morphosyntax, together with vocabulary, and reading comprehension. This reflects the multicausal and complex nature of reading impairments.

In relation to reading comprehension, the level of oral language was its greatest predictor. This relationship showed until which degree the affectation that children with DLD present in the morphosyntactic component will have an impact on their reading comprehension. It is important to highlight that the limitations of children with DLD in reading of pseudo-words imply an obstacle to read new words. This is the result of a bidirectional relationship between language and reading. Thus, whether children with DLD have problems learning new words, their lexicon will be restricted, which have repercussions on their reading comprehension skills (Cain and Oakhill, 2006).

After proving the connection between oral language and reading skills in children with DLD, this study is meant to identify and characterize the influence of socioeconomic, educational, and family variables on the development of their oral language and literacy skills. It is widely recognized that oral and reading skills are highly sensitive to socioeconomic conditions. Variables such as the mothers' education level and the amount of income are generally similar in most countries where evidence has been obtained (Huttenlocher et al., 2002). Nonetheless, since the Colombian socioeconomic conditions are inequitable, mothers with a higher educational level do not necessarily have better income. This study analyzed the first variable independently. Thus, the results indicate that there is a stronger relationship between the mothers' educational level and the development of reading skills as well as with the level of income received.

This same effect was observed in the level of oral language. Previous studies have demonstrated that the educational level of the mothers is determinant in the development of children's language (Hoff and Tian, 2005). From this perspective, it is viable to identify some routes which explain these differences. For example, those mothers with a better educational level will probably develop emerging literacy practices related to alphabet knowledge or shared reading; those practices are directly linked to receptive and expressive language skills of their children. This fact is coherent with some research performed in this field (Weigel et al., 2006).

Within the study, family support given to the participants in their academic tasks was also analyzed. The research found strong relationships between this variable and oral language and reading skills. Moreover, if the mothers' educational level is considered along with family's support an effect on the reading level of the participants. This could imply that mothers who have a better educational level become more involved in their children's education and assume an active role that is directly related to later literacy processes such as reading. This finding is interesting, since family support added to a good educational level could enrich children's linguistic environment and thereby reduce the negative effects that DLD brings on school performance.

By observing the relationship among children with DLD, their pairs in the control group, and the types of school they attend to (public vs. private); it was not possible to identify significant differences. This suggests that the type of school is not a protective factor in relation to the academic development of children with DLD. It is well known that children who study in a private school usually come from families with higher income, which is not a significant factor either. The mother's educational level and the family's support are the variables that truly determine a difference in both oral language and reading development.

Academic grade average was another variable analyzed within the study. According to the results, this variable has direct connections with oral language and reading levels (especially accuracy and comprehension). Likewise, significant inter-group differences were found, which suggests that children with a higher academic grade average tend to present better levels of oral language. This gives results being consistent with previous research performed in children with DLD (Asimina and Aikaterini, 2015; Aguilar-Mediavilla et al., 2019). They found they obtained significantly lower scores and a higher rate of grade repetition than their peers. This fact was also confirmed in this stud evidencing the reliance of oral language on subsequent literacy skills, especially in reading, which is a transversal skill to all areas of knowledge.

The repetition factor included in this study also allows hypothesizing that children with DLD may need more time to finish school. Such disadvantage can increase the likeliness of rejection by their parents, which makes the gap between one and other bigger; thus, the “Matthew Effect” is restated in these children. Future studies might explore these connections through longitudinal studies that identify long term effects at the emotional and social participation levels of children with DLD in educational contexts. In the same way, it is relevant to observe what happens when schoolwork becomes more complex, for instance, when children with DLD reach high school, where reading demands are greater and reading comprehension requires other skills.

Despite the relevance of the findings in this study, there were some limitations related to the number of children with DLD. However, this population is underdiagnosed in the Colombian context, and this interferes with the early identification of these children, which is the reason why the results found must be carefully analyzed without reaching generalizations for the entire population. In addition, the study did not have data of differentiated grades by subject or measures related to the socio-affective profile of the participants, elements that would have been key in the analysis and should be considered in depth in future studies.

Finally, this study has several clinical and educational implications. First, language impairments transcend beyond early childhood, which have direct consequences in literacy processes of children with DLD at school. This, added to a lower performance and a higher repetition factor, makes necessary to implement direct actions that support continuity and success of this population in educational contexts. Moreover, as it was discussed, the mother's educational level is more related to the oral language and reading levels in children with DLD than the socioeconomic status. That is the reason why the mother's educational level becomes a protective factor for academic processes. Likewise, the phonemic level of phonological awareness is directly linked to accuracy and reading of new words; hence, a direct training at this level may benefit reading skills of children with DLD. Furthermore, the oral language level, specifically the morphosyntactic knowledge, shows direct relationships with reading comprehension processes, which highlight the bidirectional connection between oral language and reading skills.

## Conclusions

Oral language skills are crucial in the subsequent development of reading. Spanish speaking children with DLD have oral language difficulties that go past early literacy because reading accuracy and comprehension skills are affected; this has several repercussions like a lower academic average and a higher repetition factor, that directly interferes in their academic context. Nonetheless, not all language impairments impact reading in the same way as the morphosyntactic level directly impacts comprehension, whereas the phonological skills directly impact accuracy.

The evidence found is consistent with the “Matthew Effect” since the impairments of children with lower performances in language affect the development of reading. In this sense, children with this profile, present a higher risk of having academic difficulties than their peers, which would be even higher if there is no family support, or the mother has a low educational level.

## Data Availability Statement

The original contributions presented in the study are included in the article/supplementary material, further inquiries can be directed to the corresponding author/s.

## Ethics Statement

The studies involving human participants were reviewed and approved by Research Ethics Committee of the Faculty of Medicine, Universidad Nacional de Colombia. Written informed consent to participate in this study was provided by the participants' legal guardian/next of kin.

## Author Contributions

MFLD, AMM, and JCBR: conceptualization and writing—review and editing. JCBR: formal analysis. AMM: investigation and writing—original draft preparation. MFLD: supervision, review, and editing. All authors contributed to the article and approved the submitted version.

## Conflict of Interest

The authors declare that the research was conducted in the absence of any commercial or financial relationships that could be construed as a potential conflict of interest.

## Publisher's Note

All claims expressed in this article are solely those of the authors and do not necessarily represent those of their affiliated organizations, or those of the publisher, the editors and the reviewers. Any product that may be evaluated in this article, or claim that may be made by its manufacturer, is not guaranteed or endorsed by the publisher.
